# Effect of Microbial Fermentation on the Fishy-Odor Compounds in Kelp (*Laminaria japonica*)

**DOI:** 10.3390/foods10112532

**Published:** 2021-10-21

**Authors:** Wenyang Zhu, Bo Jiang, Fang Zhong, Jingjing Chen, Tao Zhang

**Affiliations:** 1 State Key Laboratory of Food Science and Technology, Jiangnan University, Wuxi 214122, China; 6190111170@stu.jiangnan.edu.cn (W.Z.); fzhong@jiangnan.edu.cn (F.Z.); jingjinc@jiangnan.edu.cn (J.C.); zhangtao@jiangnan.edu.cn (T.Z.); 2 International Joint Laboratory on Food Science and Safety, Jiangnan University, Wuxi 214122, China; 3 School of Food Science and Technology, Jiangnan University, Wuxi 214122, China

**Keywords:** kelp (*Laminaria japonica*), odor activity value, fermentation, fishy odor, gas chromatography-ion mobility spectrometry, gas chromatography-mass spectrometry

## Abstract

Kelp (*Laminaria japonica*) is an important marine resource with low cost and rich nutrition. However, its fishy odor has compromised consumer acceptance. In this study, the effects of fermentation with *Lactobacillus plantarum* FSB7, *Pediococcus pentosaceus* CICC 21862 and *Saccharomyces cerevisiae* SK1.008 on fishy notes in kelp was studied using gas chromatography-mass spectrometry (GC-MS), gas chromatography-ion mobility spectrometry (GC-IMS) and odor activity values (OAVs). Forty-four volatile organic compounds (VOCs) were identified in unfermented kelp, most of which were aldehydes, followed by alkanes, alcohols and ketones. Among them were 19 volatile compounds with OAV greater than one. Substances containing *α*,*β*-unsaturated carbonyl structure (1-Octen-3-one, (*E*,*Z*)-2,6-nonadienal, (*E*,*E*)-2,4-decadienal, etc.) are the main contributors to kelp fishy odor. The number of VOCs in kelp samples fermented by *L. plantarum*, *P. pentosaceus* and *S. cerevisiae* were decreased to 22, 24 and 34, respectively. GC-IMS shows that the fingerprint of the *S. cerevisiae* fermented sample had the most obvious changes. The disappearance of 1-octen-3-one and a 91% decrease in unsaturated aldehydes indicate that *S. cerevisiae* was the most effective, while *L. plantarum* and *P. pentosaceus* only reached 43–55%. The decrease in kelp fishy notes was related to the decrease in *α*,*β*-unsaturated carbonyl groups. The experimental results show that odor reduction with fermentation is feasible.

## 1. Introduction

Depending on different pigmentation, seaweeds are divided into red algae, green algae and brown algae [1]. Kelp (*Laminaria japonica*) belongs to brown algae and is rich in nutrients such as iodine, protein, alginate, glycol, amino acids and polyunsaturated fatty acids [2]. Fucoidan, which is rich in kelp, has immune modulating, antioxidant and anti-inflammatory effects [3]. Therefore, kelp is a “longevity food” widely consumed in East Asia [4]. The world’s leading seaweed producers are China, Indonesia and the Philippines. These three countries also have the largest number of seaweed species and the longest history of seaweed consumption [5]. In addition to being used for the production of hydrocolloids and in agriculture, most of the harvested seaweeds are used for food (75%) [6]. However, the global market share of seaweed aquaculture production for food and other uses is still less than 1% of the total biomass production [3,7].

The typical fishy odor of kelp is one of the main obstacles to its use as a bulk ingredient. Odor-causing compounds are found in many foods and can impede consumption. The beany flavor in soybeans or peas is mainly caused by aldehydes (hexanal, (*E*)-2-hexenal) and alcohols (hexanol, 1-octen-3-ol) [8]. (*Z*)-6-nonenal, (*E*,*Z*)-2,6-nonadienal, octanol, hexanal, and 6-methyl-5-hepten-2-ol contributed to the thermally sensitive aroma of fresh watermelon [9]. Ma et al. [10] found that 2-acetyl-1-pyrroline and alcohols contributed to the aroma of cooked rice; aldehydes, benzene derivatives and acids were the causes of flavor deterioration during storage. Thus far, there are few studies on the characterization of fishy odor compounds from kelp. Seo et al. [11] argue that isovaleric acid, allyl isothiocyanate, octanal and acetaldehyde are the main contributors to kelp odors. Studies by Takahashi et al. [12] have shown that 1-iodooctane, nonanal, (*E*)-2-nonenal, (*E*,*Z*)-2,6-nonadienal and 1-octen-3-ol are the vital main components of kombu (*Laminaria* spp.) odor.

Thus far, some studies have reported physical and chemical methods such as cyclodextrin embedding and flower tea cover-up to remove the fishy odor of kelp [13,14]. These means of odor removal are mainly adsorption and masking of fishy odor. However, these treatments may require special processing means and are not easily controlled [15]. The flavor of the final product could be suppressed and even introduction of other undesirable flavors could occur. As a result, the acceptability of processed kelp was decreased.

Biological deodorization is currently a reliable and efficient method. Fermentation can be used to improve the flavor of food and reduce unpleasant odors. Nedele et al. [16] investigated aroma changes by fermenting soy drink with *Lycoperdon pyriforme*. After 28 h fermentation, the reduction in green odorants (hexanal, (*E*)-2-nonenal, (*E*,*E*)-2,4-decadienal) was consistent with the sensory difference. According to the study of Yi et al. [17], fermentation by *Lactobacillus plantarum* could effectively remove the beany flavor components (1-octen-3-ol, hexanal and hexanol) of mung beans. After confirming that (*E*)-2-nonenal and (*E*,*E*)-2,4-decadienal are key aroma compounds of wheat bread crumb, Vermeulen et al. [18] used *S. cerevisiae* and *Lactobacillus sanfranciscensis* for fermentation under different conditions, and found that the transformation pathway was different. Facts have proved that microorganisms can metabolize certain ingredients in food, thereby changing the ingredients and affecting their flavor characteristics. At present, there are two main types of microorganisms used in food fermentation: yeast and lactic acid bacteria [19]. They have a wide range of fermentation applicability and good fermentation effect.

Based on the above research, the purpose of this study was to analyze the volatile organic compounds (VOCs) of kelp, find out its fishy-odor contributors, and evaluate the effects of fermentation on the fishy odorants of kelp. More importantly, the specific reasons for deodorization can be inferred by comparing the fermentation effects of different strains. This provides a basic reference for further research on the mechanism of deodorization of kelp in the future. Gas chromatography-mass spectrometry (GC-MS) and gas chromatography-ion mobility spectrometry (GC-IMS) were used to analyze VOCs of different kelp samples. The odor activity value (OAV) is equal to the ratio of the concentration of each compound to the odor threshold, which was used to identify the substances that played an important role in kelp odor. At last, the kelp was fermented by yeast (*Saccharomyces cerevisiae* SK1.008) and lactic acid bacteria (*Lactobacillus plantarum* FSB7 and *Pediococcus pentosaceus* CICC 21862) to compare the deodorization effects. The results of this study may contribute to understanding the changes in fishy-odor compounds in kelp, as well as providing important implications for kelp processing.

## 2. Materials and Methods

### 2.1. Materials

Fresh kelp (*Laminaria japonica*) was harvested from a kelp farm (Shandong Haizhibao ocean technology Co., Ltd., Weihai, Shandong, China). The raw samples were salted and vacuum-packed, transported to the laboratory and stored at −20 °C. Salted kelp was washed three times to remove salt and impurities. After drying the surface and selecting healthy thin tissue, kelp was cut into about 1 × 1 cm square pieces. The final samples were stored in screw-capped glass flasks at 4 °C in the dark for no longer than 3 days until the next step.

### 2.2. Microbial Fermentation

*L. plantarum* FSB7 and *S. cerevisiae* SK1.008 were obtained from our laboratory. *P. pentosaceus* CICC 21862 was purchased from the China Center of Industrial Culture Collection (CICC, Beijing, China).

*S. cerevisiae* SK1.008 was inoculated on Yeast Extract Peptone Dextrose Medium (YPD; 10 g/L Yeast Extract, 20 g/L Peptone, 20 g/L Dextrose) agar, then the strain was grown in YPD broth at 35 °C for 18 h. Then the strain was centrifuged and diluted to 10^6^ CFU/mL. The *S. cerevisiae* suspension was used as the starter, added 1% into the conical flask with the mass ratio of kelp to the water of 1:3 and fermented at 35 °C and 150 rpm for 6 h. The sample fermented by yeast *S. cerevisiae* was called YF. In contrast, unfermented kelp samples were called UF.

The *L. plantarum* FSB7 and *P. pentosaceus* CICC 21862 were cultured in MRS broth at 37 °C for 16–24 h then centrifuged and resuspended in sterilized water to obtain 10^8^ CFU/mL. Inoculated into a conical flask under the same conditions as above. Inoculated samples were fermented at 37 °C in an incubator for 8 h. The samples fermented by *L. plantarum* and *P. pentosaceus* were called LF and PF, respectively.

### 2.3. Headspace-Solid Phase Microextraction (HS-SPME) Conditions

A manual SPME holder equipped with 1 cm 50/30 μm divinylbenzene/carboxen/polydimethylsiloxane fiber (Supelco, Bellefonte, PA, USA) was conditioned at GC injector at 250 °C for 30 min before extraction. The final HS-SPME process contained the following steps: 5 g sample was placed in 20 mL vial attached with a top hole-cap with a PTFE/silicone septum (Shendi Glass Instrument Co., Ltd., Shanghai, China) then put on a magnetic stirrer (RCT Basic, IKA, Aachen, Germany). After being incubated at 60 °C degrees for 15 min, the SPME fiber was exposed to the kelp headspace for 30 min for extraction and then desorbed in the GC injection port at 250 °C for 5 min under splitless conditions.

### 2.4. GC-MS Analysis

Finnigan Trace GC ultra-chromatograph with a TSQ Quantum XLS mass detector (Thermo Fisher Scientific, Waltham, MA, USA) equipped with a fused polar capillary column (DB-WAX, 30 m × 0.25 mm × 0.25 µm; J&W Scientific, Folsom, CA, USA) was used.

Instrument parameters were set in reference to López-Pérez et al. [20]. The oven temperatures were programmed starting at 50 °C for 2.5 min, followed by increases to 90 °C at 3 °C/min, 140 °C at 3.5 °C/min, 180 °C at 5 °C/min, 240 °C at 15 °C/min, and finally was held at 240 °C for 10 min for column cleanliness. Helium was used as a carrier gas with a constant flow of 1 mL/min. The MS detector was operated in the full scan mode at 70 eV electron ionization, data were collected at 1.74 scans/s over the m/z range of 33 to 300 amu. A series of C7-C40 n-paraffins (Sigma-Aldrich, St. Louis, MO, USA) were injected to obtain retention index (RI).

The mass spectra of the collected aroma substances were compared with the NIST 11 and Wiley mass spectral libraries to identify volatile compounds. Additionally, the RI was compared with the reported literature, and 2,4,6-Trimethylpyridine (10 μL, 4.585 μg/mL in anhydrous alcohol) was used as an internal standard for semi-quantitative analysis.

### 2.5. OAV Calculation

The contribution of each volatile compound was usually evaluated by OAV; it was calculated according to

(1) $$\mathrm{OAV}=\frac{C}{\mathrm{OT}}$$

where C is the concentration of the volatile compound and OT represents the odor threshold reported in the literature. Compounds with a threshold higher than 1 were considered to have significant effects on aroma composition. For compounds with multiple thresholds, the most recent data were selected.

### 2.6. Headspace-Gas Chromatography-Ion Mobility Spectrometry (HS-GC-IMS) Analysis

A GC-2010 gas chromatograph (Shimadzu, Kyoto, Japan) equipped with a DB-WAX capillary column (60 m × 0.25 mm × 0.25 µm) and IMS instrument (FlavourSpec^®^, Gesellschaft für Analytische Sensorsysteme mbH, Dortmund, Germany) was used.

Each kelp sample (5 g) was placed into a 20 mL headspace vial and incubated at 60 °C for 15 min. Then, 1 mL of headspace gas was injected into the injector (80 °C, splitless mode) utilizing a heated syringe at 65 °C. Oven temperature started at 50 °C for 2.5 min, followed by increasing to 90 °C at 3 °C/min, 180 °C at 4 °C/min, 230 °C at 20 °C/min and finally was held at 230 °C for 15 min. The ionization source of the IMS was tritium ^3^H which provided radiation energy of 6.5 KeV. The ions were placed into a drift tube (9.8 cm length) through a shutter grid, which was operated at constant voltage (500 V/cm) and temperature (45 °C). The drift gas (nitrogen) flow was set at a constant flow rate of 150 mL/min. All analyses were performed in triplicate. N-ketones C4–C9 (Sinopharm Chemical Reagent Beijing Co., Ltd., Beijing, China) were used to calculate the RI of volatile compounds. The qualitative analysis of volatile compounds was conducted based on the GC-IMS and NIST database built in GC × IMS Library Search.

### 2.7. Data Analysis

For GC-MS, raw data were acquired using Xcalibur software (version 1.4 SR1-Thermo Fisher Scientific, Inc.). For GC-IMS, semi-quantitative and qualitative analysis was completed by Laboratory Analytical Viewer (LAV) software and all images were generated by Gallery Plot plug-in. The cluster heat map and the principal component analysis (PCA) were produced by Origin 2018 software. One-way analysis of variance (ANOVA) with Duncan’s multiple range test was carried by SPSS Statistics 26 (IBM Corporation, Armonk, NY, USA). The significance of difference was set as *p* < 0.05. The results are presented as means ± standard deviation (SD).

## 3. Results and Discussion

### 3.1. HS-SPME-GC-MS Analysis

#### 3.1.1. Identification of Volatile Compounds in Kelp

Volatile compounds in kelp were detected using HS-SPME-GC-MS. A total of 44 VOCs (Table 1) could be classified into eight families, which include eighteen as aldehydes, nine as alcohols, seven as ketones, one as furan, two as esters, four as halogens, two as alkanes and one as alkene. The GC profiles of the different kelp samples can be seen in the Supplementary Data (Figure S1). López-Pérez et al. [21] studied the VOCs of seven species of seaweed, most of which were coincident with ours. As shown in Table 1, aldehydes, particularly unsaturated aldehydes were the most abundant compounds, followed by alcohols and ketones.

Regarding the aldehydes family, hexanal, (*E*)-2-octenal, (*E*)-2-nonenal and nonanal were most abundant, this result was similar to Ferraces-Casais et al. [22]. Alcohols came next in content to aldehydes, 1-octen-3-ol was the most abundant alcohol in kelp, and after that were (*E*)-2-octen-1-ol and 2-nonen-1-ol. Takahashi, et al. [12] identified volatile compounds of dried kombu (*Laminaria* spp.) by GC-MS and GC-sniffing, and they believe that nonanal, (*E*)-2-heptenal, (*E*)-2-octenal, (*E*)-2-octen-1-ol and 1-octen-3-ol constitute the kombu odor. The ketones detected in the kelp were mainly trans-*α*-ionone, (*E*,*E*)-3,5-octadien-2-one and 1-octen-3-one. Ketones usually have a distinctive odor. The ionones are 13-carbon compounds with a violet and woody aroma. Found in many fruits and flowers, they are products of the oxidative 9,10 bond-degradation of carotenoids [23]. The high levels of carotenoids in kelp produce a large amount of *α*-ionone, resulting in the unique floral fragrance of kelp.

#### 3.1.2. Identification of Volatile Compounds in Fermented Kelp

As shown in Table 1, the number of VOCs in three fermentation samples decreased. Compounds in yeast *S. cerevisiae* SK1.008 fermented kelp sample (YF) were decreased to 33 species with the number of aldehydes greatly reduced, and alcohols became the most abundant compounds. Yeast metabolism can produce higher alcohols; they are considered to be a family of aroma compounds [24]. Some specific amino acids, such as proline and leucine, can increase the corresponding production of higher alcohols such as 3-methylbutol, which were not detected before. In YF, most of the aldehydes disappeared, and (*E*)-2-nonenal and (*E*)-2-octenal decreased by about 90%. Polyunsaturated aldehydes are known to be degradation products of unsaturated fatty acids. Again, trans-á-ionone was still the most abundant of the ketones in YF. Certain odorous ketones, such as (*E*,*E*)-3,5-octadien-2-one and 1-octen-3-one were missing; instead, 3-octanone and 2,3-octanedione were produced.

Volatile substances in *P. pentosaceus* CICC 21862 fermented kelp (PF) were also greatly reduced, but the content of aldehydes was still high. Hexanal was 1.85 times compared with that in the unfermented sample, while the content of nonanal, octanal, heptanal and (*E*)-4-heptenal showed different degrees of increase. The signal intensity of 1-octen-3-one, trans-á-ionone and 6-methyl-5-hepten-2-one were weak. Enals and enones usually have unpleasant odors, and given that their levels do not drop much, it is clear that PF still has a strong seaweed fishy odor. When it comes to *L. plantarum* FSB7 fermented kelp (LF), the results were almost the same with PF.

### 3.2. OAV Analysis of Key Fishy-Odor Compounds in Kelp

The odor descriptions, odor thresholds and corresponding OAV were listed in Table 2. We arranged by OAV of unfermented kelp, where an OAV greater than one indicates that the substance contributes to the composition of kelp odor profile. There were 19 substances in UF with an OAV greater than one.

At the top of the list is 1-octen-3-one, because it has a high content, a low threshold (0.01 μg/kg) and also the smell of metallic, mushroom, dirt. Therefore, it can be postulated that 1-octen-3-one is the main component of the fishy odor of kelp. This ketone is also known to be the degradation product of unsaturated fatty acids by chemical autoxidation reactions [27,28]. A unique phenomenon in the YF sample is that the disappearance of 1-octen-3-one is accompanied by the generation of 3-octanone, which is consistent with the results of Wanner and Tressl [28] and La Guerche et al. [29]. In their study, reductases extract of *S. cerevisiae* irreversibly catalyzing the enantioselective reduction of *α*,*β*-unsaturated carbonyl and the conversion ratio of 1-octen-3-one catalyzed to 3-octanone reached 90%. It was later confirmed that it was enone reductases or enoate reductases [EC 1.3.1.31]. They predominantly belonged to the “Old Yellow Enzyme” family of flavin and NADPH-dependant reductases [30]. This is why many aldehydes and ketones containing *α*,*β*-unsaturated carbonyl in YF samples have been reduced and their contents were significantly decreased. 1-Octen-3-ol is another contributor; it has a fatty, grass and mushroom flavor. Studies have shown that 1-octen-3-ol can be derived from the oxidative decomposition of linoleic acid with 10-hydroperoxide [31].

Unsaturated aldehydes (*E*,*Z*)-2,6-nonadienal, (*E*,*E*)-2,4-decadienal and (*E*)-2-nonenal ranked second, third and fifth. The 2-alkenals and 2,4-alkadienals have extremely low odor thresholds, so they play an important role in the overall odor profile. Aldehydes usually have a green, fatty, tallow odor; in sensory science, these flavors are called aldehyde flavors. With the increase in C-chain length, the odor threshold decreases and the odor becomes less citrusy, more fat-like [32]. These aldehydes are mainly produced by lipid oxidation, which may be related to a large number of lipids in kelp. According to literature reports, lipoxygenase in soybeans and peas catalyze unsaturated fatty acids into aldehydes [33]. In cereals, unsaturated fatty acids are oxidized by lipoxygenase during crushing or grinding, and then decomposed into aldehydes [34]. The decrease in the content of unsaturated aldehydes was also due to the effect of enone reductases.

Substances containing *α*,*β*-unsaturated carbonyl structure, such as 1-octen-3-one, 2,4-nonadienal, cis-4,5-epoxy-(*E*)-2-decenal, 1-hepten-3-one, (*E*)-2-decenal and 2-undecenal were significantly reduced in YF, which indicated that the fishy-odor compounds were sharply reduced. The odor profiles of LF and PF were similar. The content of most of the substance content was basically unchanged. In general, the content of fishy odorants decreased slightly. This may be related to the content and activity of enone reductase in yeast and lactic acid bacteria.

### 3.3. HS-GC-IMS Analysis

**Figure 1:**
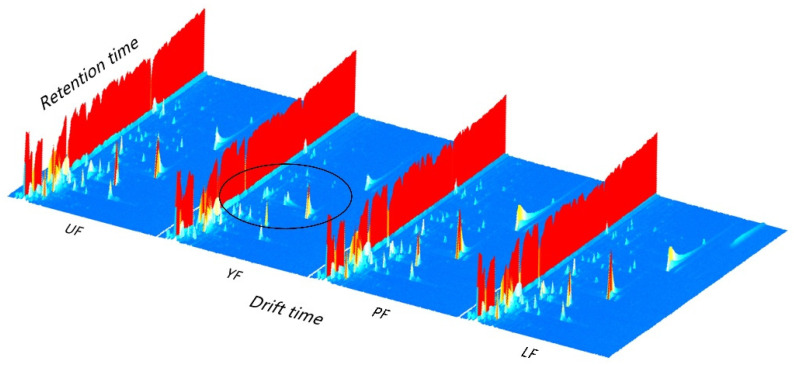
Gas chromatography-ion mobility spectrometry (GC-IMS) 3D topographic plot of four different treatments of kelp samples. UF: unfermented kelp; YF: yeast *S. cerevisiae* fermented kelp; PF: *P. pentosaceus* SK1.008 fermented kelp; LF: *L. plantarum* FSB7 fermented kelp.

Figure 1 is a 3D topographical visualization of volatile compounds of kelp in four different treatments (UF, YF, PF, LF). The ordinate represents the retention time of the gas chromatograph, the abscissa indicates the ion drift time after normalization and the *Z*-axis shows the intensity of the peak. The color depth of the point means the concentration of the substance, from blue to red, representing the concentration is getting higher [35]. In Figure 1, YF’s aroma compounds were significantly different from other samples, and the changing substances are circled.

The qualitative and quantitative results of the volatile compounds in different treatment kelp samples were shown in Table 3. Eighty-two volatile compounds from four different treatments kelp samples were listed. Most of them were identified by the GC-IMS NIST database, but there were still 13 compounds with no qualitative results due to the limited data from the library database. It was worth mentioning that some volatile compounds were repetitive, which was their monomer and dimer with similar retention time and different drift times [36].

**Figure 2:**
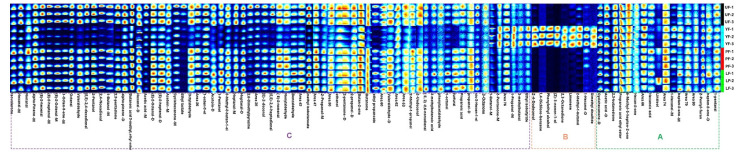
Gallery plots (fingerprints) of volatile compounds in four different kelp samples. M: monomer; D: dimer. (**A**–**C**) regions represent different volatile organic compounds (VOCs) content characteristics of UF, YF, PF and LF.

Figure 2 is divided into three areas. Region A represents the VOCs shared by the four samples with similar contents. Area B represents the substances with more contents in YF, and contains the following substances: dimethyl disulfide,1-hexanol-D, 3-methyl-1-butanol, limonene, 2,3-octanedione and others. Among these substances, alcohols are the most common compounds. Alcohols are produced by the oxidation and decomposition pathways of grease and lipid, and 3-Methyl-1-butanol is the side product from the alcohol fermentation of starch and sugar. Ge et al. [37] also found that the content of 3-methyl-1-butanol was significantly increased in hot drying peppers. The thermal oxidation of polyunsaturated fatty acids or the thermal degradation of amino acids produced ketones. Fan et al. [34] and Chen et al. [38] reported an increase in 2-pentanone and 2,3-octanedione in their samples after cooking or fermentation. Additionally, 3-Methylbutanal belongs to Strecker aldehyde. The reaction to form Strecker aldehydes is one of a series of complex reactions, collectively referred to as the Maillard reaction [39]. In region C, there are the following substances: hexanal, nonanal, (*E*)-2-octenal, (*E*,*E*)-2,4-decadienal, octanal, phenylacetaldehyde, etc. Most of them were odor-causing compounds, with a harsh, green, unpleasant odor. The content of volatile substances in YF is significantly less than that of the other three. This result is consistent with GC-MS OAV analysis: enone reductases or enoate reductases catalyze the reduction of *α*,*β*-unsaturated carbonyl structure.

In addition, there were substances not detected by GC-MS: 2,6-Dimethylpyrazine is one of the alkyl pyrazines, and it has a musty, tobacco, earthy mushroom odor. However, its threshold is high, its OAV value is low and it has little effect on the overall odor. Pyrazines and their derivatives play an important role in food aroma. Several reports have highlighted the microbial origin of pyrazine in fermented soybeans and cheese [40]. Alpha-Pinene and limonene both belong to the terpenes. Terpenes are a group of natural hydrocarbons that are widely found in plants [41]. Linalool is a kind of terpene alcohol. It is synthesized from the *α*-pinene or *β*-pinene contained in turpentine.

### 3.4. Analysis Based on PCA Results and Heat Map Clustering

**Figure 3:**
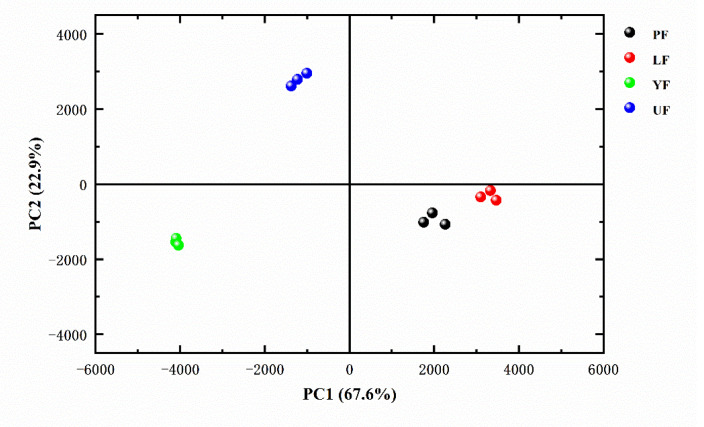
Principal component analysis (PCA) scores plot based on the signal intensity obtained from four different kelp samples.

A total of 12 sets of signal intensity data from four samples from GC-IMS were processed, dimensionally reduced and the PCA was carried out (Figure 3). These irrelevant variables obtained through the PCA can reflect the main information of the original variables. The first two principal components (67.6 and 22.9% of PC1 and PC2, respectively) explained 90.5% of the total variance, indicating that the two principal components could reveal most information of different samples. In Figure 3, four samples are well separated, forming 3 regions, which indicates that it is very good to distinguish the differences between the samples. PF and LF had similar positive and negative score values, so they had similar odor profiles. This indicated that the lactic acid bacteria had similar effects on kelp fermentation.

**Figure 4:**
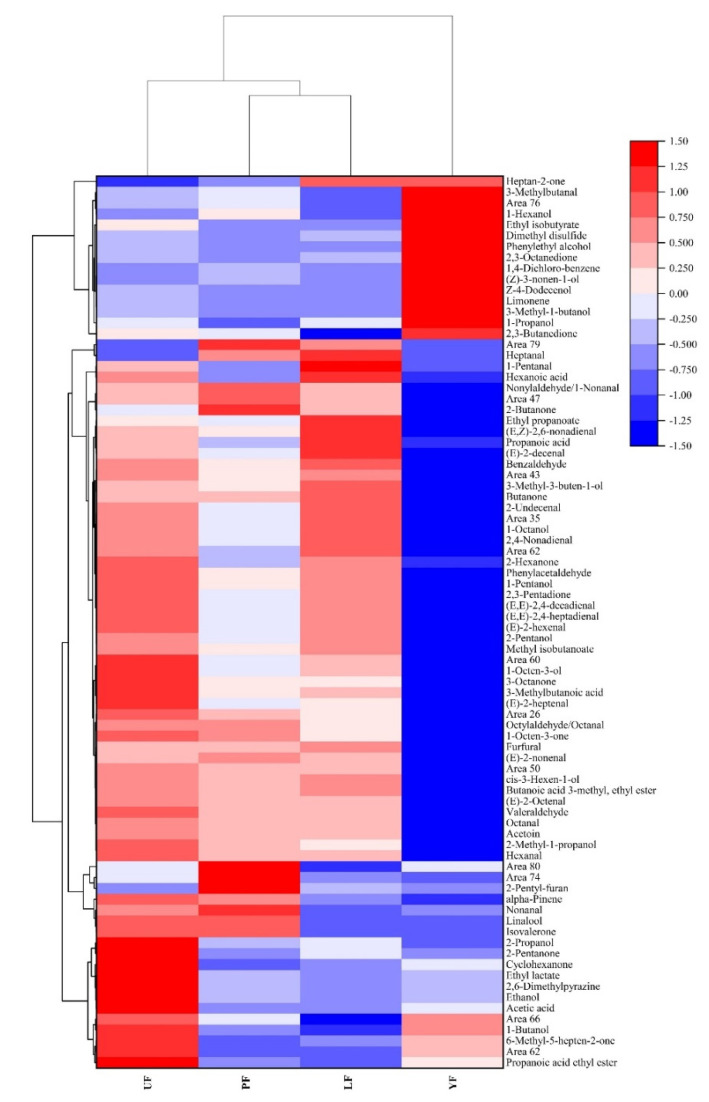
Heat map clustering in volatile flavor compounds of kelp samples.

To further study the odor profile between the kelp of different fermentation groups, heat map clustering analysis is used, which can clearly and intuitively reflect the differences. The signal strength of each volatile flavor is marked with a different color on the heat map (Figure 4). From bottom to top is blue to white and last to red, which indicates increasing peak signal intensity. PF and LF samples clustered together, which means that they had the highest correlation. Based on the vertical mode, the volatile compounds in kelps were classified into four groups according to the peak intensity. In the bottom two areas, the content of 2-propanol, 2-pentanone, 2,6-dimethylpyrazine, etc., were distributed differently in four samples due to their odor difference. The middle part of the heat map contains substances such as 1-octen-3-one, 1-octen-3-ol and many aldehydes which have unpleasant odors such as green, fatty, pungent and tallow. The compounds at the top have a mild, rose-like, buttery and fruity odor with a low odor threshold. Therefore, the YF sample had fewer fishy odors and more fruit and flower aromas.

## 4. Conclusions

In this study, we studied the effect of microbial fermentation on the odor of kelp. Forty-four volatile compounds in unfermented kelp were detected by GC-MS. The most abundant substances were aldehydes, followed by alkanes, then alcohols and ketones. OAV results showed that unsaturated aldehydes and ketones such as 1-octen-3-one, (*E*,*Z*)-2,6-nonadienal, (*E*,*E*)-2,4-decadienal and (*E*)-2-nonenal, were the main contributors to the fishy odor of kelp. They contain *α*,*β*-unsaturated carbonyl structures, often with extremely low odor thresholds and unpleasant smells. After microbial fermentation, the odor profile changed significantly, which can be directly seen from the GC-IMS fingerprint. Yeast *S. cerevisiae* SK1.008 had a critical impact; the VOCs were diminished to 34, 1-octen-3-one had vanished and the levels of aldehydes had a steep drop, whereas *L. plantarum* FSB7 and *P. pentosaceus* CICC 21862 do not have such a strong deodorization performance. The different degrees of reduction in fishy odor may be related to the effect of enone reductase in different microorganisms in catalyzing the reduction of unsaturated bonds. This demonstrates that the strategy of microbial fermentation can influence the odor profile of kelp and can even diminish the fishy odor and make strides in the worthiness. The combined use of GC-MS and GC-IMS maximizes the results by combining accurate analytical results with intuitive visualization. Further research should focus on sensory-directed flavor analysis to clarify the flavor of kelp more thoroughly and comprehensively. At the same time, the study of the degradation pathways of fishy-odor compounds by *S. cerevisiae* are also important.

## Figures and Tables

**Table 1:** Volatile compounds identified in different treatment kelp samples by gas chromatography-mass spectrometry (GC-MS).

No.	Compound	Rt ^a^ (min)	RI ^b^		Concentration (μg/kg)			
			Cal ^c^	Ref ^d^	UF	YF	PF	LF
	Aldehydes							
1	Hexanal	4.46	1087	1089	35.56	5.01	65.87	65.34
2	(*E*)-2-pentenal	5.46	1133	1130	2.19	ND **^e^**	ND	4.38
3	Heptanal	6.36	1182	1182	8.11	ND	8.32	12.68
4	(*E*)-2-hexenal	7.45	1212	1214	ND	ND	7.44	7.74
5	(*E*)-4-heptenal	7.63	1239	1243	1.87	ND	2.52	ND
6	Octanal	8.66	1283	1284	7.88	2.19	12.23	8.43
7	(*E*)-2-heptenal	9.54	1319	1321	20.21	ND	ND	ND
8	Nonanal	11.19	1385	1385	27.39	8.29	49.47	28.0
9	(*E*)-2-octenal	12.40	1417	1425	96.32	11.65	58.26	41.14
10	(*E*,*E*)-2,4-heptadienal	13.87	1478	1476	6.19	ND	ND	ND
11	Benzaldehyde	14.85	1512	1513	12.39	ND	7.66	ND
12	(*E*)-2-nonenal	15.12	1574	1573	126.76	10.38	55.79	48.60
13	(*E*,*Z*)-2,6-nonadienal	16.51	1574	1576	40.25	2.18	9.17	4.15
14	(*Z*,*Z*)-3,6-nonadienal	16.91	1589	1591	2.15	ND	ND	ND
15	(*E*)-2-decenal	17.86	1625	1625	15.16	ND	ND	ND
16	2,4-Nonadienal	19.51	1687	1686	14.27	ND	0.198	ND
17	2-Undecenal	20.97	1753	1755	10.86	ND	ND	ND
18	(*E*,*E*)-2,4-decadienal	21.37	1758	1758	94.75	5.07	23.04	14.41
19	cis-4,5-Epoxy-(*E*)-2-decenal	27.10	1997	2000	7.96	ND	ND	ND
	Alcohols							
1	3-Methyl-1-butanol	7.22	1201	1201	ND	90.11	ND	ND
2	1-Hexanol	10.22	1346	1345	37.55	57.19	ND	ND
3	(*Z*)-3-hexen-1-ol	11.02	1370	1372	ND	0.69	ND	ND
4	1-Octen-3-ol	13.18	1457	1455	67.87	34.80	10.28	11.43
5	1-Heptanol	13.66	1451	1453	ND	23.42	ND	ND
6	2-Ethyl-1-hexanol	14.02	1485	1486	78.63	ND	ND	ND
7	1-Phenyl-1-decanol	15.44	1518	-	ND	ND	ND	3.52
8	1-Octanol	15.69	1543	1540	29.54	35.80	ND	ND
9	1-Nonen-3-ol	16.01	1554	1555	ND	0.34	ND	ND
10	(*Z*)-2-octen-1-ol	17.32	1604	1605	53.07	13.76	4.85	ND
11	1-Nonanol	18.41	1646	1653	50.39	40.06	ND	ND
12	*Z*-4-Dodecenol	18.52	1630	-	1.14	10.69	ND	ND
13	*Z*-2-Dodecenol	19.15	1652	-	0.74	ND	6.80	4.71
14	(*Z*)-3-nonen-1-ol	19.60	1687	1688	ND	25.22	ND	ND
15	(*E*)-2-nonen-1-ol	19.96	1704	1703	84.99	ND	ND	ND
16	(*E*)-6-nonen-1-ol	19.97	1710	1714	ND	9.30	ND	ND
17	1-Decanol	21.01	1760	1763	ND	3.04	ND	ND
18	(*Z*)-5-decen-1-ol	24.31	1886	-	ND	10.69	ND	ND
19	Phenylethyl alcohol	26.25	1928	1932	ND	20.25	ND	ND
	Ketones							
1	3-Octanone	8.05	1248	1248	ND	14.46	ND	ND
2	1-Octen-3-one	8.98	1296	1295	39.57	ND	32.43	29.48
3	1-Hepten-3-one	9.15	1303	1303	2.41	ND	ND	3.16
4	2,3-Octanedione	9.68	1326	1325	ND	9.83	ND	ND
5	6-Methyl-5-hepten-2-one	9.79	1329	1330	2.94	2.52	2.10	ND
6	4-Octen-3-one	10.57	1360	-	1.19	ND	ND	ND
7	(*E*,*E*)-3,5-octadien-2-one	16.18	1562	1562	108.44	ND	ND	ND
8	(*E*)-6,10-dimethyl-5,9-undecadien-2-one	23.39	1848	1849	19.81	ND	ND	ND
9	trans-á-Ionone	25.5	1926	1926	204.63	35.06	28.38	17.37
	Halogens							
1	1-Iodo-propane	2.89	956	965	4.27	ND	ND	ND
2	1-Iodo-pentane	5.42	1137	1164	14.64	13.26	7.03	9.43
3	3-Bromo-pentane	9.50	1317	-	13.65	ND	39.0	30.88
4	1-Iodo-heptane	10.33	1350	1384	4.11	ND	6.76	3.55
5	1,4-Dichloro-benzene	13.1	1452	1450	ND	17.47	ND	ND
	Alkanes							
1	Tetradecane	14.01	1408	-	108	ND	ND	ND
2	Pentadecane	14.12	1494	1500	334.44	27.42	30.10	20.16
	Furan							
1	2-Pentyl-furan	7.20	1220	1222	24.31	12.60	17.58	13.52
	Alkenes							
1	1,4-Octadiene	22.88	1799	-	7.81	ND	ND	ND
2	1-Tridecyne	23.87	1873	-	ND	8.33	ND	ND
3	5-Ethyl-1-nonene	24.58	1899	-	ND	15.47	ND	ND
	Esters							
1	1-Octen-3-ol-acetate	10.75	1367	-	3.10	ND	ND	ND
2	Nonanoic acid, methyl ester	14.92	1492	1491	13.71	ND	ND	ND
3	Decanoic acid, methyl ester	17.01	1597	1599	ND	9.68	ND	ND
	Acid							
1	Oxalic acid	2.66	937	-	ND	31.31	ND	ND
	Benzene derivative							
1	p-Cymene	7.98	1271	1268	ND	ND	6.81	5.84

^a^ The retention time of volatile compounds on DB-Wax columns. ^b^ The retention index (RI). ^c^ The retention index was calculated against n-alkanes C7-C40 on DB-Wax columns. ^d^ Reference RI (DB-WAX column) were published on NIST Chemistry WebBook (https://webbook.nist.gov/chemistry/; accessed on 26 August 2021) and ChemSpider (http://www.chemspider.com/; accessed on 28 August 2021). “-” means there is no RI in the library. ^e^ Not detected in the sample. UF means unfermented kelp; YF means *Saccharomyces cerevisiae* fermented kelp; PF means *Pediococcus pentosaceus* SK1.008 fermented kelp; LF means *Lactobacillus plantarum* FSB7 fermented kelp.

**Table 2:** The odor activity values (OAVs) and odor description of volatile compounds detected in kelp samples.

No. ^a^	Compound	Odor Description ^b^	Odor Threshold ^c^ (μg/kg)	OAV			
				UF	YF	PF	LF
1	1-Octen-3-one	metallic, mushroom, dirt	0.01	3957.2	0	3242.8	2948
2	(*E*,*Z*)-2,6-nonadienal	cucumber, cucumber peel, green, green leaves	0.02	2012.7	109.1	458.6	207.7
3	(*E*,*E*)-2,4-decadienal	fatty, green, wax, aldehyde, deep fried, fried fat, oily	0.07	1353.6	72.5	329.1	205.8
4	trans-á-Ionone	cedar, floral, artificial raspberry, cooked carrots, violet	0.6	341.1	58.4	47.3	28.9
5	(*E*)-2-nonenal	bast, cucumber, fatty, green, oxidized, stale, tallow	0.4	316.9	26	139.5	121.5
6	2,4-Nonadienal	deep fried fat, fatty, fried potato, oily, soapy	0.05	285.4	0	39.6	0
7	cis-4,5-Epoxy-(*E*)-2-decenal	green, metallic	0.13	61.2	0	0	0
8	1-Hepten-3-one	fatty, fruity, grass	0.04	60.2	0	0	78.9
9	(*E*)-2-decenal	fatty, green	0.3	50.5	0	0	0
10	1-Octen-3-ol	fatty, fruity, grass, mushroom, raw mushrooms, sweet	1.5	45.2	23.2	6.9	7.6
11	(*E*)-2-octenal	almond, fatty, fruity, green, nutty, burdock, tallow	3	32.1	3.9	19.4	13.7
12	Nonanal	citrus, fatty, floral, green grass, pungent, soapy, tallow	1.1	24.9	7.5	45	25.5
13	2-Undecenal	aldehyde, metallic, green	0.78	13.9	0	0	0
14	Octanal	aldehyde, fatty, fruity, orange peel, pungent, soapy	0.9	8.8	2.4	13.6	9.4
15	Hexanal	aldehyde, grass, green, leaves, vinous	5	7.1	1	13.2	13.1
16	2-Pentyl-furan	fruity, green grass	6	4.1	2	2.9	2.3
17	Heptanal	fatty, green, heavy, oily, putty	3	2.7	0	2.8	4.2
18	(*E*)-2-heptenal	fatty, fruity, green, melting plastic, soapy, tallow	13	1.6	0	0	0
19	1-Nonanol	green, sweet, oily	45.5	1.1	0.9	0	0
20	(*Z*)-2-octen-1-ol	fatty, rancid	75	0.7	0.2	0.1	0
21	(*E*)-2-nonen-1-ol	green, waxy melon	130	0.7	0	0	0
22	(*E*,*E*)-3,5-octadien-2-one	sweet, balsamic, vanilla, dill hay, oxidized	150	0.7	0	0	0
23	(*E*,*E*)-2,4-heptadienal	fatty, nutty	15.4	0.4	0	0	0
24	(*E*)-6,10-dimethyl-5,9-undecadien-2-one	floral, fruity, fatty, green, pear, apple, banana nuances	60	0.3	0	0	0
25	(*E*)-4-heptenal	dairy, biscuit, cream, fatty, fishy, sweet	10	0.2	0	0.3	0
26	1-Octanol	green herbaceous	130	0.2	0.3	0	0
27	1-Hexanol	fatty, floral, green	250	0.2	0.2	0	0
28	2-Ethyl-1-hexanol	mild oily, sweet, slightly floral	1280	0.1	0	0	0
29	6-Methyl-5-hepten-2-one	citrus, mushroom, pepper, rubber, strawberry	50	0.1	0.1	0	0
30	(*E*)-2-pentenal	almond, apple, green	55	0	0	0	0.1
31	Benzaldehyde	almond, bitter almond	350	0	0	0	0
32	(*E*)-2-hexenal	almond, bitter, green, heavy, stinkbug	17	0	0	0.4	0.5
33	Z-2-dodecenol	-	41	0	0	0.2	0.1
34	3-Methyl-1-butanol	fruity, banana, sweet, fragrant, powerful	250	0	0.4	0	0
35	(*Z*)-3-halchexen-1-ol	green, grass	200	0	0	0	0
36	1-Heptanol	herb	330	0	0.1	0	0
37	(*E*)-6-nonen-1-ol	powerful, melon, green	1	0	9.3	0	0
38	1-Decanol	floral odor, orange flowers	47	0	0.1	0	0
39	Phenylethyl alcohol	rose-like, bitter, sweet, peach	60	0	0.3	0	0
40	3-Octanone	earthy, ethereal, ketone, mushroom, resinous	23	0	0.6	0	0
41	1-Octen-3-ol-acetate	lavender, metallic, mushroom-like	90	0	0	0	0
42	Decanoic acid, methyl ester	fruity odor, grape	12	0	0.8	0	0
43	p-Cymene	citrusy aroma, lemon	0.01	0	0	681.5	584.2

^a^ Rank in descending order according to the OAV values of UF. ^b^ Odor description refers to *Fenaroli’s Handbook of Flavor Ingredients* [25] and The LRI and Odour Database (http://www.odour.org.uk/; accessed on 26 August 2021). ^c^ Odor threshold in water. The values were according to the reported and *Compilations of odour threshold values in air, water and other media* [26].

**Table 3:** Volatile compounds identified in different kelp samples by Gas chromatography-ion mobility spectrometry (GC-IMS).

No.	Compound	DT ^a^ (ms)	RT ^b^ (s)	RI ^c^	Comment	Signal Intensity			
						UF	YF	PF	LF
1	Propanoic acid ethyl ester	1.1391	386.4	968.7		17,479.39 ± 1024	16,576.75 ± 167.44	16,524.81 ± 280.28	16,501.87 ± 518.37
2	Isovalerone	1.8044	704.8	1169.4		9009.6 ± 227.5a	5606.09 ± 32.26a	8790.98 ± 368.81b	5251.53 ± 141.78b
3	Hexanal	1.5578	563.6	1096.8	monomer	8524.21 ± 230.13a	3730.53 ± 97.72c	7197.59 ± 194.32b	7510.7 ± 301.53b
	Hexanal	1.2572	573.4	1010.1	dimer				
4	Nonanal	1.9686	1042	1387.1		5197.54 ± 285.31b	2884 ± 49.46c	5830.12 ± 259.32a	2462.1 ± 63.24d
5	alpha-Pinene	1.2863	434.2	1000	monomer	3617.51 ± 180.87a	2491.84 ± 111.99b	3376.24 ± 175.6a	2741.81 ± 99.86b
	alpha-Pinene	1.6701	443.8	989.7	dimer				
6	Acetic acid	1.1521	1193.8	1447.7	monomer	3520.98 ± 141.86a	2050.12 ± 61.5b	1603.4 ± 24.57c	1477.26 ± 44.32c
	Acetic acid	1.0503	1195	1443.2	dimer				
7	Methyl isobutanoate	1.1438	306.2	933.4		3057.95 ± 107.75a	875.24 ± 25.49c	2440.84 ± 87.13b	3115.91 ± 48.24a
8	Linalool	2.2208	1363.2	1530.4		2279.88 ± 103.93a	1526.01 ± 31.04c	2135.22 ± 32.51b	1395.21 ± 16.33d
9	1-Butanol	1.3595	561.6	1106.9	monomer	1812.33 ± 61.59a	1686.54 ± 50.19b	1383.21 ± 13.83c	1263.44 ± 70.66d
	1-Butanol	1.9607	575	1107.2	dimer				
10	2,3-Pentadione	1.3063	481.8	1062.3		1283.54 ± 26.19a	301.81 ± 4.67d	892.47 ± 13.59c	1082.67 ± 45.74b
11	2-Pentanone	1.1246	318.6	932.6	monomer	1259.46 ± 32.67a	920.75 ± 5.33b	940.09 ± 43.7b	984.95 ± 46.74b
	2-Pentanone	1.1279	459	924.3	dimer				
12	Acetoin	1.2489	824.6	1230.7	monomer	1042.84 ± 40.15a	497.18 ± 7.47c	964.55 ± 5.55b	980.27 ± 40.56b
	Acetoin	1.2692	954		dimer				
13	2-Butanone	1.0591	306.8	911.6		1012.42 ± 60.74ab	942.8 ± 33.22b	1070.13 ± 39.01a	1051.2 ± 22.03a
14	2-Pentyl-furan	1.8771	845	1234.1		1017.81 ± 31.3b	1014.2 ± 20.7b	1321.08 ± 53.75a	1073.31 ± 18.41b
15	Ethanol	1.1128	318.6	943.6		1023.83 ± 25.68a	696.59 ± 17.59b	679.46 ± 6.73b	619.39 ± 10.84c
16	2,3-Butanedione	1.1772	472.8	991		967.24 ± 14.53b	1067.87 ± 26.78a	899.04 ± 21.04c	744.24 ± 22.23d
17	Area 80	2.043	1040.2			918.01 ± 23.18b	920.59 ± 9.11b	992.1 ± 43.33a	817.96 ± 24.82c
18	Valeraldehyde	1.1838	438.4	997.5	monomer	872.69 ± 8.73a	320.44 ± 4.91c	759.89 ± 13.03b	764.33 ± 27.02b
	Valeraldehyde	1.4196	435.2	999.8	dimer				
19	(*E*)-2-Octenal	1.3299	1156.6	1443.8	monomer	881.88 ± 25.04a	402.72 ± 16.11c	771.04 ± 15.42b	785.5 ± 32.5b
	(*E*)-2-Octenal	1.8139	1153	1445.7	dimer				
20	Heptanal	1.3259	741.2	1197.3	monomer	757.72 ± 19.59c	794.05 ± 15.57c	1532.95 ± 40.16b	1689.16 ± 61.52a
	Heptanal	1.6328	735.2	1198.6	dimer				
21	Ethyl isovalerate	1.2592	562.8	1088.1		829.7 ± 20.81a	539.7 ± 13.95b	807.02 ± 22.91a	821.44 ± 36.92a
22	Area 79	1.3336	562			822.54 ± 24.96c	820.22 ± 21.21c	1063.23 ± 28.41a	1003.36 ± 38.63b
23	2-Pentanol	1.2869	629.8	1129.1		805.2 ± 24.16a	282.82 ± 5.99c	620.66 ± 12.41b	832.07 ± 16.32a
24	Butanone	1.0596	325.8	957		787.6 ± 30.51a	395.99 ± 15.84c	733.39 ± 35.25b	835.75 ± 17.51a
25	(*E*)-2-hexenal	1.5131	808.4	1233.8		781.79 ± 35.64a	218.19 ± 9.81d	529.04 ± 23.53c	710.73 ± 7.18b
26	(*E*)-2-heptenal	1.6662	993	1338.8	monomer	677.47 ± 23.95a	318 ± 11.84d	490.28 ± 21.41c	553.1 ± 11.25b
	(*E*)-2-heptenal	1.2518	996.2	1337.2	dimer				
27	(*E*,*E*)-2,4-decadienal	1.3576	1154.4	1815.7		678.43 ± 48.78	213.86 ± 3.78	486.83 ± 19.23	652.38 ± 32.62
28	3-Methyl-3-buten-1-ol	1.1785	812	1260.2		693.59 ± 26.87b	322.38 ± 3.22d	641.06 ± 10.99c	721.52 ± 18.66a
29	Area 62	1.0758	489.2			610.11 ± 24.82a	545.76 ± 9.36b	410.72 ± 8.69c	408.34 ± 22.41c
30	Octanal	1.4094	985.8	1294.1		562.22 ± 8.56a	207.95 ± 6.24c	501.38 ± 8.6b	519.83 ± 23.52b
31	Cyclohexanone	1.4496	946.4	1312.5	monomer	516.94 ± 28.5a	457.05 ± 14.06b	439.72 ± 11.41b	436.1 ± 11.65b
	Cyclohexanone	1.1517	947.2	1313.2	dimer				
32	Area 47	1.139	484.6			500.01 ± 30.15a	345.08 ± 3.45b	524.37 ± 20.19a	501.73 ± 25.09a
33	6-Methyl-5-hepten-2-one	1.0887	1002.2	1340.2		500.86 ± 9.82a	454.41 ± 22.72b	396.64 ± 11.26c	414.56 ± 16.7c
34	2-Propanol	1.0859	293.4	930.8	monomer	474.44 ± 23.96a	328.65 ± 7.39c	360.02 ± 23.35bc	374.03 ± 12.31b
	2-Propanol	1.084	295.2	933.8	dimer				
35	2-Hexanone	1.1888	490.4	1054.2		485.91 ± 18.94a	427.86 ± 11.06b	439.5 ± 17.1b	480.86 ± 16.83a
36	1-Octen-3-one	1.6756	948.2	1315.7		447.93 ± 17.98a	147.85 ± 2.54d	417.78 ± 11.86b	366.69 ± 12.83c
37	Area 74	1.7416	448.4			369.09 ± 8.76b	344.49 ± 5.28c	446.71 ± 12.06a	347.29 ± 14.37c
38	1-Octen-3-ol	1.1552	1171	1454.4		368.35 ± 16.55a	303.28 ± 4.57c	327.73 ± 8.28b	359.54 ± 9.24a
39	1-Pentanol	1.2483	819.6	1252.4		360.63 ± 22.33a	132.63 ± 2.74d	290.18 ± 5.69c	323.29 ± 14.38b
40	1-Propanol	1.2611	437.8	1011.7	monomer	457.04 ± 10.7b	557.02 ± 20.74a	423.87 ± 4.86c	466.59 ± 14.16b
	1-Propanol	1.1098	433	1031.7	dimer				
41	2,6-Dimethylpyrazine	1.1352	1020.6	1351.7		293.16 ± 10.26a	132.41 ± 3.61b	121.36 ± 2.73c	91.12 ± 2.68d
42	3-Methyl-1-butanol	1.243	773	1204.1		265.2 ± 6.7b	1022.17 ± 26.26a	122.45 ± 1.88c	108.4 ± 3.08d
43	Area 62	1.1294	516.4			264.03 ± 4.05b	184.45 ± 2.86d	227.66 ± 3.87c	274.86 ± 3.13a
44	(*E*,*E*)-2,4-heptadienal	1.1434	1193.7	1482.4		259.04 ± 11.52a	101.31 ± 2.37c	198.92 ± 4.21b	247.99 ± 11.14a
45	Hexanoic acid	1.3107	1768.2	1863.4		263.23 ± 5.26b	221.44 ± 2.57c	228.61 ± 9.65c	279.05 ± 4.79a
46	Area 50	1.117	351.6			237.68 ± 4.75a	143.47 ± 5.84b	231.95 ± 2.3a	230.7 ± 5.83a
47	Area 43	1.1886	1261			209.37 ± 8.78b	119.39 ± 1.18d	195.49 ± 3.04c	224.38 ± 8.17a
48	Z-4-Dodecenol	1.4889	1258.2	1996.2		221.7 ± 10.69a	1523.34 ± 39.9a	126.52 ± 4.01c	113.03 ± 4.77c
49	2,4-Nonadienal	1.6144	1197.4	1668.5		219.26 ± 8.66a	91.12 ± 2.29c	161.72 ± 2.48b	220.32 ± 5.95a
50	Octylaldehyde	1.3974	932	1299.3		188.96 ± 4.87a	79.05 ± 0.92c	186.55 ± 11.19a	161.65 ± 7.31b
51	Heptan-2-one	1.6268	733.8	1189.6	monomer	190.74 ± 1.1d	247.64 ± 2.5a	203.41 ± 4.15c	253.52 ± 3.82a
	Heptan-2-one	1.2591	731.2	1191.4	dimer				
52	1-Hexanol	1.6343	1030	1405.1	monomer	186 ± 8.94d	479.07 ± 5.5a	293.7 ± 14.88b	167.54 ± 6.53c
	1-Hexanol	1.3215	1028.8	1405.7	dimer				
53	Area 26	1.5704	951.4			179.22 ± 5.38a	94.59 ± 3.85d	163.4 ± 7.41b	148.26 ± 3.88c
54	Ethyl lactate	1.5308	1022.4	1358.3		178.42 ± 7.33a	46.25 ± 1.51b	45.9 ± 0.69b	40.43 ± 0.86b
55	(*E*)-2-nonenal	1.404	1321.4	1562.3		169.76 ± 4.58b	102.36 ± 1.54c	178.34 ± 2.72a	176.83 ± 5.02a
56	(*E*)-2-decenal	1.2193	1401.6	1647.8		176.05 ± 2.99b	91.82 ± 2.82c	143.27 ± 3.72c	198.84 ± 5.37a
57	Area 35	1.1726	1011.8			167.62 ± 4.32b	115.66 ± 1.8d	151.63 ± 6.74c	181.77 ± 9.45a
58	Benzaldehyde	1.1473	1316.4	1547		166.39 ± 8.32a	123.56 ± 3.37c	151.77 ± 5.53b	173.95 ± 8.38a
59	3-Methylbutanoic acid	1.2172	1548.8	1688.5		157.99 ± 4.64a	69.03 ± 3.45d	125.03 ± 5.62c	134.01 ± 3.3b
60	Nonylaldehyde	1.4688	1099.8	1386.7		161.1 ± 3.33b	64.07 ± 1.5d	193.09 ± 9.65a	158.94 ± 4.77c
61	2-Undecenal	1.1014	1451.6	1755.8		160.57 ± 6.34b	78.66 ± 2.04d	121.01 ± 4.41c	173.91 ± 6.21a
62	(*E*,*Z*)-2,6-nonadienal	1.3679	1405	1590.3		151.41 ± 3.72b	84.88 ± 2.41d	140.03 ± 1.41c	178.14 ± 8.91a
63	cis-3-Hexen-1-ol	1.2591	1143	1433.4		148.1 ± 5.95a	99.57 ± 1.54b	143.21 ± 3.51a	150.29 ± 5.86a
64	Area 66	1.0893	380.4			140.43 ± 9.2a	137.76 ± 2.7a	130.92 ± 2.71ab	125.55 ± 2.55b
65	Area 76	1.2492	853.4			127.03 ± 1.49b	172.74 ± 7.84a	133.15 ± 5.55b	117.58 ± 1.83c
66	Ethyl isobutyrate	1.3225	379.8	983.5		121.49 ± 2.08b	159.34 ± 2.79a	100.94 ± 1.52c	100.49 ± 6.52c
67	Phenylacetaldehyde	1.2622	1435.4	1648.5		104.34 ± 4.76a	51.12 ± 0.79d	82.9 ± 0.84c	96.85 ± 2.49b
68	2-Methyl-1-propanol	1.1375	502	1101		93.02 ± 4.56a	51.65 ± 0.91c	85.89 ± 4.71b	82.31 ± 2.22b
69	Area 60	1.1018	347.6			94.71 ± 6.81a	45.78 ± 1.61d	68.68 ± 1.07c	81.83 ± 2.21b
70	Propanoic acid	1.1028	1232	974.5		91.15 ± 3.32b	56.64 ± 1.92d	75.83 ± 0.86c	110.37 ± 4.57a
71	3-Methylbutanal	1.4013	379	920.8		87.88 ± 1.32b	124.45 ± 3.83a	89.1 ± 1.78b	71.72 ± 2.56c
72	Furfural	1.3234	1245.6	1472.6		65.17 ± 3.26a	18.91 ± 0.49b	67.18 ± 1.96a	67.19 ± 1.04a
73	1-Octanol	1.4643	1319	1566.6		61.05 ± 0.93a	38.02 ± 0.96c	53.09 ± 0.31b	64.42 ± 3.88a
74	Limonene	1.2283	770.8	1212.1		45.15 ± 0.7b	159.16 ± 4.12a	21.51 ± 0.67c	22.17 ± 0.25c
75	3-Octanone	1.3285	925.4	1272.8		43.05 ± 1.62a	26.46 ± 0.68c	38.35 ± 1.92b	37.23 ± 1.67b
76	(*Z*)-3-nonen-1-ol	1.1696	1399.6	1685.7		42.92 ± 0.43bc	506.88 ± 8.69a	47.93 ± 2.09b	37.75 ± 0.22d
77	Ethyl propanoate	1.1427	348	911.3		41.12 ± 0.98b	14.73 ± 0.17c	38.26 ± 0.78b	63.35 ± 3.05a
78	1-Pentanal	1.2098	318	925.5		36.2 ± 1.47b	33.92 ± 0.68c	34.44 ± 0.52bc	38.91 ± 0.8a
79	1,4-Dichloro-benzene	1.4674	1199.8	1450		31.81 ± 0.83c	193.32 ± 1.95a	34.18 ± 0.86b	28.83 ± 0.57d
80	2,3-Octanedione	1.1697	1202.7	1325.6		24.49 ± 0.87bc	144.76 ± 4.34a	21.5 ± 1.5c	26.84 ± 0.41b
81	Phenylethyl alcohol	1.4656	1801.5	1915.2		20.54 ± 0.42b	211.13 ± 3.18a	16.22 ± 0.48c	17.55 ± 0.43bc
82	Dimethyl disulfide	1.1977	584	1111.1		12.38 ± 0.3b	40.59 ± 0.47a	11.41 ± 0.42c	11.83 ± 0.54bc

^a^ Retention indexes; ^b^ retention times; ^c^ drift times. Different letters (a, b, c, d) show significant differences at a 95% confidence level.

## Supplementary Materials

The following are available online at https://www.mdpi.com/article/10.3390/foods10112532/s1, Figure S1: Gas chromatography profiles of different kelp samples. UF: unfermented kelp; YF: yeast *Saccharomyces cerevisiae* fermented kelp; PF: *Pediococcus pentosaceus* SK1.008 fermented kelp; LF: *Lactobacillus plantarum* FSB7 fermented kelp.
