# Supplementary material for: Effect of Microbial Fermentation on the Fishy-Odor Compounds in Kelp (Laminaria japonica)
Source: Foods. 2021 Oct 21;10(11):2532. doi: 10.3390/foods10112532 (PMC8623561; doi:10.3390/foods10112532)

Supplemental Data

Figure S1: Gas chromatography profiles of different kelp samples. UF: unfermented kelp; YF: yeast *Saccharomyces cerevisiae* fermented kelp; PF: *Pediococcus pentosaceus* SK1.008 fermented kelp; LF: *Lactobacillus plantarum* FSB7 fermented kelp.

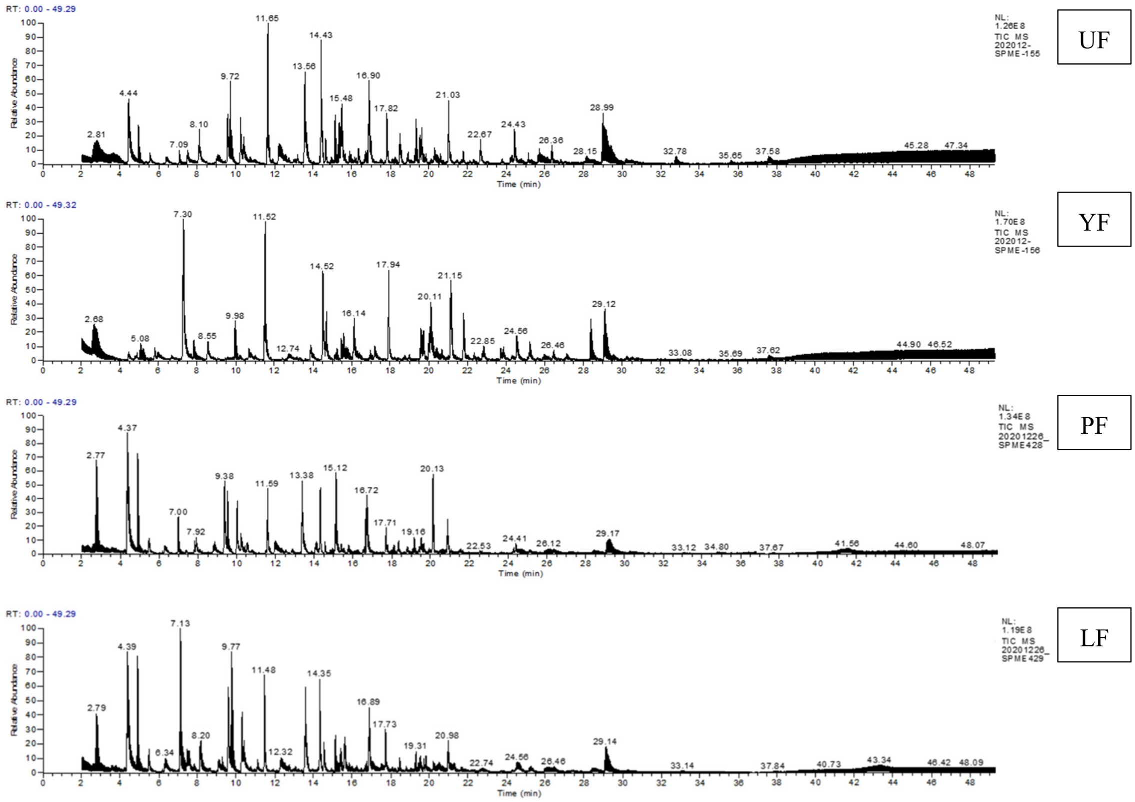

Supplement: Supplementary file 1 [file foods-10-02532-s001.zip › foods-1386281-supplementary.pdf]
